# Supplementary material for: Ferrielectricity controlled widely-tunable magnetoelectric coupling in van der Waals multiferroics
Source: Nat Commun. 2024 Apr 8;15:3029. doi: 10.1038/s41467-024-47373-7 (PMC11001967; doi:10.1038/s41467-024-47373-7)
Supplement: Supplementary file 1 — Supplementary Information [file 41467_2024_47373_MOESM1_ESM.pdf]

Supplementary Information: Ferroelectricity controlled widely-tunable magnetoelectric coupling in van der Waals multiferroics

Qifeng Hu,[1,*] Yuqiang Huang,[1,*] Yang Wang,[2,*] Sujuan Ding,[3,*] Minjie Zhang,[1,*] Chenqiang Hua,[1] Linjun Li,[4,†] Xiangfan Xu,[5] Jinbo Yang,[6] Shengjun Yuan,[7] Kenji Watanabe,[8] Takashi Taniguchi,[8] Yunhao Lu,[1,†] Chuanhong Jin,[3,†] Dawei Wang,[2] and Yi Zheng[1,†]

[1]*School of Physics, and State Key Laboratory of Silicon and Advanced Semiconductor Materials, Zhejiang University, Hangzhou 310027, China*

[2]*Zhejiang Province Key Laboratory of Quantum Technology and Device, School of Physics, Zhejiang University, Hangzhou 310027, China*

[3]*State Key Laboratory of Silicon and Advanced Semiconductor Materials, Zhejiang University, Hangzhou 310027, China*

[4]*State Key Laboratory of Extreme Photonics and Instrumentation, College of Optical Science and Engineering, Zhejiang University, Hangzhou 310027, China*

[5]*School of Physics Science and Engineering, Tongji University, Shanghai 200092, China*

[6]*State Key Laboratory for Mesoscopic Physics, School of Physics, Peking University, Beijing, 100871, China*

[7]*School of Physics and Technology, Wuhan University, Wuhan 430072, China*

[8]*National Institute for Materials Science, 1-1 Namiki, Tsukuba, 305-0044, Japan*

Supplementary Note 1. Physical characterizations of few-layer and multilayer $CuCrP_2S_6$

High quality $CuCrP_2S_6$ (CCPS) single crystals, as confirmed by x-ray diffraction (Supplementary Fig. **1**), can be readily exfoliated down to the monolayer thickness by the standard micro-mechanical exfoliation method. Supplementary Fig.**2**a shows the optical image of a typical CCPS flake with consecutive thicknesses from monolayer to trilayer, exhibiting an unusual optical contrast enhancement from monolayer to bilayer when a Si substrate with 285 nm SiO_2 dielectric is used for sample preparations. Supplementary Fig. **2**b is the corresponding non-contact atomic force microscope (NCAFM) image of Supplementary Fig. **2**a, revealing a monolayer CCPS step height of about 0.7 nm. Layer-dependent Raman spectroscopy is summarized in Supplementary Fig. **2**c, which shows no significant peak position shift when the sample thickness is reduced from septuple-layer to monolayer.

For atomic-resolution annular dark-field scanning transmission electron microscopy (ADF-STEM), we measured multiple few-layer as well as multilayer CCPS flakes. For the bilayer sample shown in Figure 1d of the main text, the line profiling analyses (as indicated by the red line of Supplementary Fig. **3**a) clearly reveal element-sensitive contrast with the intensity ordering of $I_{\mathrm{Cu}} > I_{\mathrm{Cr}} > I_{\mathrm{P}}$ (Supplementary Fig. **3**b), in excellent agreement with the ADF-STEM simulation results.

Supplementary Note 2. Spin-filter model of bilayer CCPS

Because of the intralayer ferromagnetism and interlayer antiferromagnetic coupling, the energy barrier for opposite-spin tunnelling electrons in each CCPS ML can be treated independently. In each ML of CCPS the transmission coefficients are T_{p} and T_{ap} for tunnelling electrons with spin momentums parallel or antiparallel to the majority-spin energy barrier. For bilayer CCPS in the AFM state, the final tunnelling transmission probability is $T_{\mathrm{AFM}} = 2T_{\mathrm{p}}T_{\mathrm{ap}}$, while for the FM' state is $T_{\mathrm{FM'}} = 2(T_{\mathrm{p}}T_{\mathrm{p}} + T_{\mathrm{ap}}T_{\mathrm{ap}})$. So, the maximum magnetoresistance is $T_{\mathrm{FM'}}/T_{\mathrm{AFM}} = (T_{\mathrm{p}}T_{\mathrm{p}} + T_{\mathrm{ap}}T_{\mathrm{ap}})/T_{\mathrm{p}}T_{\mathrm{ap}}$. For $T_{\mathrm{p}} > T_{\mathrm{ap}}$, $T_{\mathrm{FM'}}/T_{\mathrm{AFM}} > 1$, which mean that the tunneling current is larger in the FM' state than that in the AFM state.

* These authors contributed equally

† e-mail: lilinjun@zju.edu.cn; luyh@zju.edu.cn; chhjin@zju.edu.cn; phyzhengyi@zju.edu.cn

Supplementary Note 3. Data processing of tunnelling magnetoresistance vs H spectroscopy

The normalized tunnelling magnetoresistance (TMR) vs magnetic field (H) data in Figure 3 of the maintext are collected as raw data of tunnelling current (I_t) vs H (Supplementary Fig. **4**a). The magnitude of I_t grows exponentially as a function of V_b setpoints due to the quantum tunnelling nature of electron transmission through CCPS MTJs. By defining $\mathrm{TMR} = [I_\mathrm{t}(H) - I_\mathrm{t}(0)]/I_\mathrm{t}(0)$, the exponential $I_\mathrm{t}(0)$ background is normalized, as shown in Supplementary Fig. **4**b. In the final step, the TMR data are further normalized by the saturation value of the FM phase, in order to highlight the V_b-dependent MEC coupling energy $\Delta E_\mathrm{MEC} \propto (H^+_\mathrm{sat} - H^-_\mathrm{sat})$. To extract the H_sat values for different V_b setpoints, we take the first derivative of normalized TMR curves and use the half-maximum points as H_sat, i.e. the valley positions of the second derivative of normalized TMR curves, as shown in Supplementary Fig. **5**.

Supplementary Note 4. Fowler-Nordheim tunnelling fitting and H_sat vs V_b characteristics of CCPS MTJs

To estimate the build-in potential introduced by the asymmetric work functions of top and bottom FLG electrodes, the $I_\mathrm{t} - V_\mathrm{b}$ characteristic is re-plotted into $|I_\mathrm{t}/V_\mathrm{b}^2|$ vs $1/V_\mathrm{b}$ for tunnelling regime analyses. By gradually increasing V_b, there will be a transition from direct tunnelling to Fowler-Nordheim tunnelling (FNT), and the latter can be expressed as [1]:

$$I_\mathrm{t} = \frac{A_\mathrm{eff} q^3 m_e V_\mathrm{b}^2}{8\pi h \phi_\mathrm{B} d^2 m_e^*} \exp[\frac{-8\pi\sqrt{2m_e^*}\phi_\mathrm{B}^{\frac{3}{2}} d}{3hqV_\mathrm{b}}], \tag{1}$$

in which d, A_eff, ϕ_B and m_e^* represent tunnelling barrier width, effective tunnelling cross-section area, barrier height and the effective electron mass, respectively, while q, m_e and h are electron charge, free electron mass and Plank's constant, respectively.

Eq. 1 can be further transformed into a linear function of $|I_\mathrm{t}/V_\mathrm{b}^2|$ and $1/V_\mathrm{b}$ by:

$$\ln\frac{I_\mathrm{t}}{V_\mathrm{b}^2} = \ln\frac{A_\mathrm{eff} q^3 m_e}{8\pi h \phi_\mathrm{B} d^2 m_e^*} - \frac{8\pi\sqrt{2m_e^*}\phi_\mathrm{B}^{\frac{3}{2}} d}{3hqV_\mathrm{b}}. \tag{2}$$

Based on Eq. 2, the onset threshold of the FNT regime can be determined by linear fitting of the low $1/V_\mathrm{b}$ data. As shown in Supplementary Fig.**6**a and **6**c for two representative

MTJs 8L-S20 and 10L-S39 respectively, the former has onset V_b of -1.4 V and 1.7 V, while the latter exhibits onset V_b of -1.45 V and 2 V. Without considering the band bending effect, the build-in potential can easily exceed 0.5 V, e.g. MTJ 10L-S39.

In the direct tunnelling regime below the FNT threshold, we are able to extract the V_b-dependent H_sat values for different $I_\mathrm{t} - V_\mathrm{b}$ characteristics. As shown in Supplementary Fig.**6**b for 8L-S20, H_sat is quasi-linearly dependent on V_b before reaching the dichotomic saturation values for different V_b polarities. Such a quasi-linear relation between H_sat and V_b is in consistency with the heterogenous ferrielectric (FiE) model proposed in the main text, which is enforced by external electric field $\mathbf{E}$ via continuous rearrangements of Cu^+ ions within the vdW cages of CrS_6 and P_2S_6 octahedrons. The quasi-linear transition of H_sat for non-saturating V_b becomes more distinctive by plotting the 2D contour of normalized $\mathrm{d}G/\mathrm{d}H$ as a function of both V_b and H. As shown in Supplementary Fig.**6**d for device 10L-S39, the 2D image unambiguously reveals two dichotomic H_sat plateaus and a linear transition area in between.

Supplementary Note 5. DFT calculations of energy potential diagrams for bilayer, trilayer and quadruple-layer CCPS

The monolayer CCPS unit-cell is consisting of two Cr atoms/UC, which is illustrated in Figure 1a of the main text. Supplementary Fig. **7**a summarizes the energy potential diagram of BL CCPS under different external electric field setpoints, taking into account the stripe-AFE ground state, the lowest metastable state meta-AFE1, and the fully polarized FE state. It is clear that the latter two states are not accessible even for an unphysical electric field of 2 V/nm. Supplementary Fig. **7**b is the energy potential diagram of trilayer CCPS, showing the AFE ground state, the lowest metastable state 3L-mAFE-I, the combination of 3L-mAFE-I and 3L-mAFE-II, and the fully polarized FE state. The lattice model representation can be readily extended to more layer numbers, e.g. there are four metastable AFE states for quadruple-layer CCPS as summarized in Supplementary Fig. **8**. It should be emphasized again that this layer-dependent rearrangements of the anti-parallel Cu^+ ions, which lead to the formation of a heterogenous FiE state, are attributed to the unique vdW depolarization effect. As summarized in Supplementary Fig. **9** for BL CCPS, DFT calculations reveal a layer-dependent charge density distribution in response to anti-

parallel Cu^+ ion displacements, which confirm that the AFE stripe domain in the bottom ML is able to have a larger displacement than the top ML.

Supplementary Note 6. $I_t - V_b$ characteristics of CCPS MTJs under different magnetic field

In complementary to the $I_t - H$ measurements under different V_b, we also perform the $I_t - V_b$ spectroscopy with fixed H setpoints. Supplementary Fig. **10**a shows two representative $I_t - V_b$ scanning loops for device 10L-S39 with $H = 0$ T and 14 T respectively. For each scanning loop, V_b is ramped by the sequence of $0 \rightarrow V_{\text{b-max}} \rightarrow -V_{\text{b-max}} \rightarrow 0$. As shown in Supplementary Fig. **10**a, both $I_t - V_b$ curves show no hysteresis or abrupt jump, proving no ferroelectric or metastable antiferroelectric phase transitions induced by external electric field $\mathbf{E}$. The full H-dependent $I_t - V_b$ characteristics are shown in linear- and log-scale in Supplementary Fig. **10**b and Supplementary Fig. **10**c respectively. All data consistently reveal a smooth evolution of $I_t - V_b$ as a function of H, with the absence of ferroelectric or metastable antiferroelectric hysteresis. Note that hysteresis behaviour emerges in the $I_t - V_b$ characteristics of thin-layer CCPS after an electrical breakdown, with a typical example shown in Supplementary Fig. **11**.

Supplementary Note 7. Derivation of detailed formulation of $\Delta E_{\mathbf{MEC}}(\mathbf{E})$

To quantitively model the FiE-interlocked magnetoelectric coupling, we define the parameter of $\Delta E_{\text{MEC}}(\mathbf{E}) = (E_{\text{FM}'}(\mathbf{E}) - E_{\text{AFM}}(\mathbf{E})) - (E_{\text{FM}'}(0) - E_{\text{AFM}}(0))$, which is a function of external electric field $\mathbf{E}$. The physical meaning of $\Delta E_{\text{MEC}}(\mathbf{E})$ is the $\mathbf{E}$-dependent energy difference of thin-layer CCPS between the AFM ground state and the FM' state. In the 1D spin-chain model[2, 3], the free energy of an AFM system with an anisotropy constant (K) and subjected to a magnetic field (H) is given by,

$$E = J_{AF} M_s^2 \cos(2\phi) + K \cos^2(\phi) - \mu_0 H M_s \cos(\phi), \tag{3}$$

where ϕ is the angle between H and the magnetic moments of the sublattice, J_{AF} ($J_{\perp}$) is the interlayer AFM coupling energy, and M_s is the net magnetic moment of an individual monolayer. Without considering the Zeeman energy, the free energy of the AFM ground

state and the momentum aligned FM' state are,

$$E_{\mathrm{AFM}} = J_{\perp} M_{\mathrm{s}}^{2} \cos(2\pi) + K \cos^{2}(\pi) = -J_{\perp} M_{\mathrm{s}}^{2} + K, \text{ and} \tag{4}$$

$$E_{\mathrm{FM'}} = J_{\perp} M_{\mathrm{s}}^{2} \cos(0) + K \cos^{2}(0) = J_{\perp} M_{\mathrm{s}}^{2} + K. \tag{5}$$

So, the total energy difference compensated by the Zeeman coupling for the AFM-FM' transition is,

$$E_{\mathrm{FM'}} - E_{\mathrm{AFM}} = 2 J_{\perp} M_{\mathrm{s}}^{2}. \tag{6}$$

It should be noted that the above relation applies only for the bulk case. For thin-layer CCPS, H_{sat} are derived by solving the $N \times N$ matrix of the 1D spin-chain model [3], and the resulting layer-dependent CAFM saturation field is:

$$H_{\mathrm{sat}} = \frac{4J_{\perp}}{\mu_0 M_{\mathrm{s}}} \cos^{2}\left(\frac{\pi}{2\mathrm{N}}\right). \tag{7}$$

Substituting Eq. 7 into Eq. 6, we get the final relation of ΔE_{MEC}:

$$\Delta E_{\mathrm{MEC}}(\mathbf{E}) = (E_{\mathrm{FM'}}(\mathbf{E}) - E_{\mathrm{AFM}}(\mathbf{E})) - (E_{\mathrm{FM'}}(0) - E_{\mathrm{AFM}}(0)) = \frac{1}{\cos^{2}(\frac{\pi}{2\mathrm{N}})} \mu_0 M_{\mathrm{s}}^{3} (H_{\mathrm{sat}}(\mathbf{E}) - H_{\mathrm{sat}}(0)). \tag{8}$$

Supplementary Note 8. Scanning Kelvin probe microscope (SKPM) of CCPS tunnelling device

Strong interfacial doping to FLG electrodes by thin-layer CCPS is evident by scanning Kelvin probe microscope, which directly measures the work functions of top and bottom FLG electrodes with an energy resolution better than 100 meV. As shown in the Supplementary Fig. **12**, the bottom (top-left corner) and top (central area) FLG electrodes exhibit distinctive work functions of about 4.6 eV and 4.3 eV, respectively. It should also be noticed that as a Dirac system, graphene is extremely sensitive to environmental doping introduced by sample preparation and device fabrications procedures. Both mechanisms contribute strong charge doing in FLG electrodes, making the work functions nearly independent on the layer number.

Supplementary Table I. Calculated average Cr-S bond lengths, Cr-S bond standard deviation, net majority spins of Cr- and S-atoms within a CrS_6 octahedron as a function of anti-parallel Cu^+ ion displacements within the FM CrS_6-P_2S_6 cages.

Δd(**Å**)	$\overline{x}$(**Å**)	$\overline{\Delta x}$(**Å**)	**Cr-d(dn)**	**S-p(dn)**	**S-p(up)**	**S-p(up-dn)**
**0 (AFE)**	2.4758	0.00443	3.385	2.680	2.815	0.135
**0.22**	2.4759	0.00509	3.396	2.681	2.798	0.117
**0.44**	2.476	0.00592	3.414	2.683	2.775	0.092
**0.65**	2.478	0.00879	3.421	2.684	2.758	0.074

[1] Lee, G.-H. *et al.* Electron tunneling through atomically flat and ultrathin hexagonal boron nitride. *Appl. Phys. Lett.* **99**, 243114 (2011).

[2] Baltz, V. *et al.* Antiferromagnetic spintronics. *Rev. Mod. Phys* **90**, 015005 (2018).

[3] Wang, Z. *et al.* Determining the phase diagram of atomically thin layered antiferromagnet $CrCl_3$. *Nat. Nanotechnol.* **14**, 1116–1122 (2019).

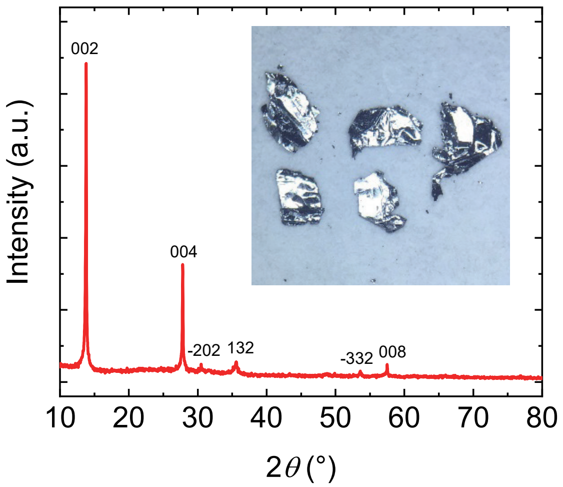

Supplementary Fig. 1. **XRD results of CCPS single crystals.** The high quality of our bulk crystals is evident by the XRD peaks of (002), (004) and (008) corresponding to the van der Waals plane. Inset is the opitc image of CCPS single crystals.

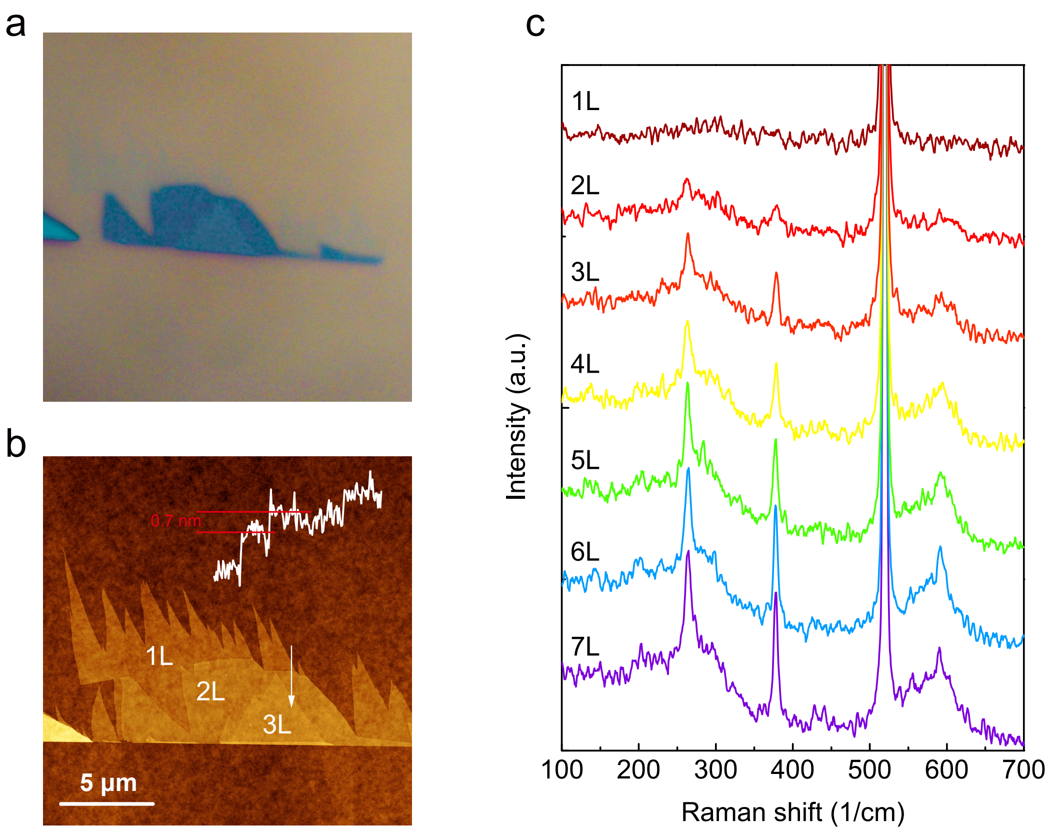

Supplementary Fig. 2. NCAFM and Raman characterizations of few-layer and multilayer CCPS flakes. **a**, Optical image of a typical CCPS flake with consecutive thicknesses from monolayer to trilayer. **b**, NCAFM image of the CCPS sample in (**a**). Using line profiling, a monolayer step height of 0.7 nm is determined. **c**, Layer-dependent Raman spectroscopy of CCPS from monolayer to 7-layers.

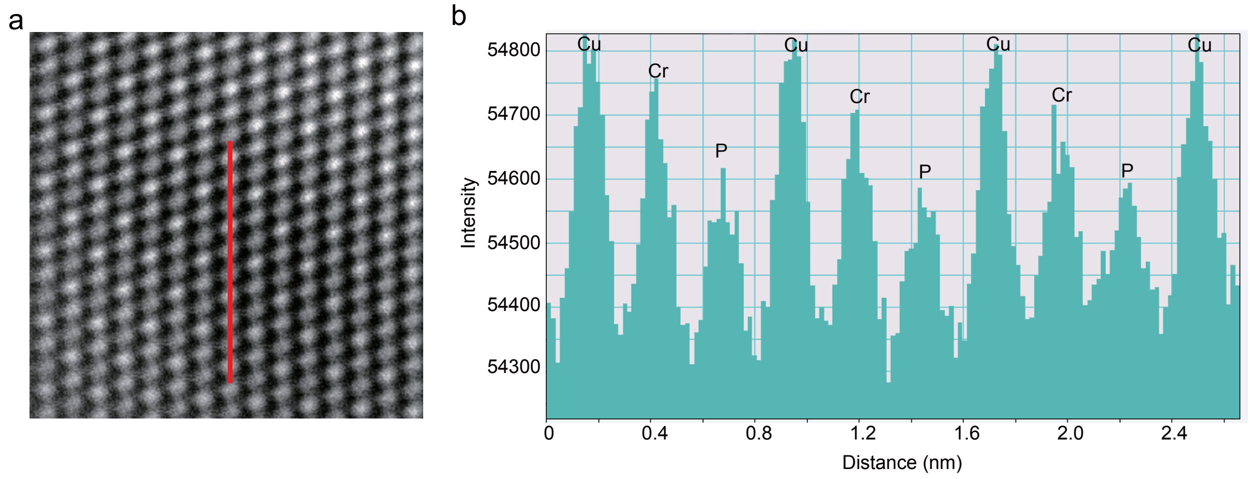

Supplementary Fig. 3. **Element analyses of the ADF-STEM images of bilayer CCPS.** **a**, ADF-STEM image of the few-layer CCPS in Figure 1d of the main text. **b**, The line profile of red line in (a). Originating in the Z-contrast, the atomic ADF-STEM intensity I is proportional to $Z^{1.x}$, where Z represents effective atomic number and 1.x is a number close to 1.5. Accordingly, the Cu, Cr and P lattice atoms can be distinguished with Cu (Z=29) exhibiting the brightest contrast, followed by Cr (Z=24), and P (Z=15) being the dimmest.

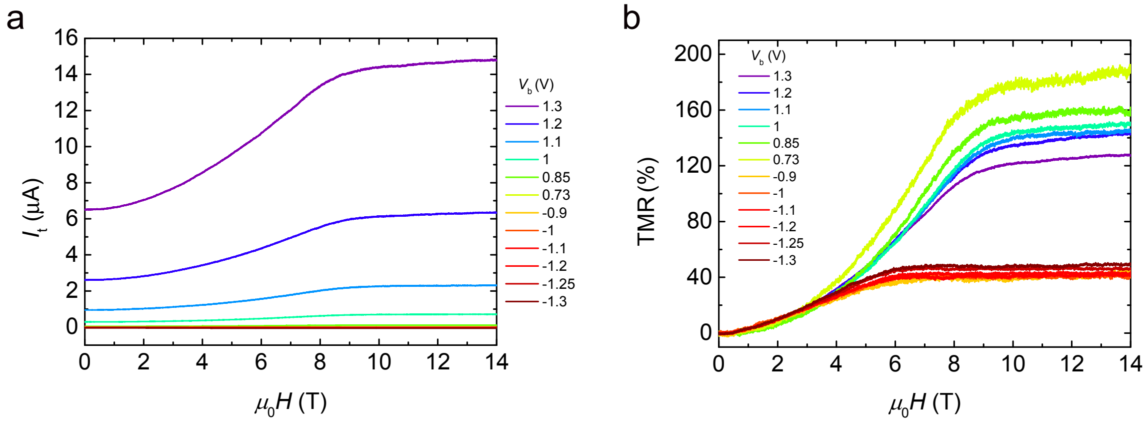

Supplementary Fig. 4. **Data processing of the CCPS MTJs.** **a**, Raw data of tunnelling current I_t vs H. **b**, Tunnelling magnetoresistance vs H, which normalizes the exponential $I_t(0)$ background.

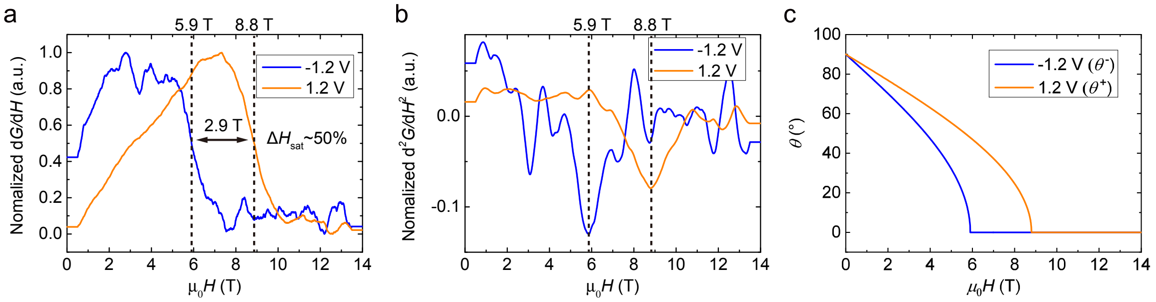

Supplementary Fig. 5. **Analyese of the CAFM saturation field $H_{\mathbf{sat}}$.** **a**, By taking the first derivative of normalized TMR curves and locating the half-maximum points, i.e. the valley positions of the second derivative of normalized TMR curves (**b**), we extract the H_{sat} values for different V_{b} setpoints. **c**, H-dependent CAFM angles for opposite V_{b}-polarity setpoints of 1.2 V (θ^{+}) and -1.2 V (θ^{-}).

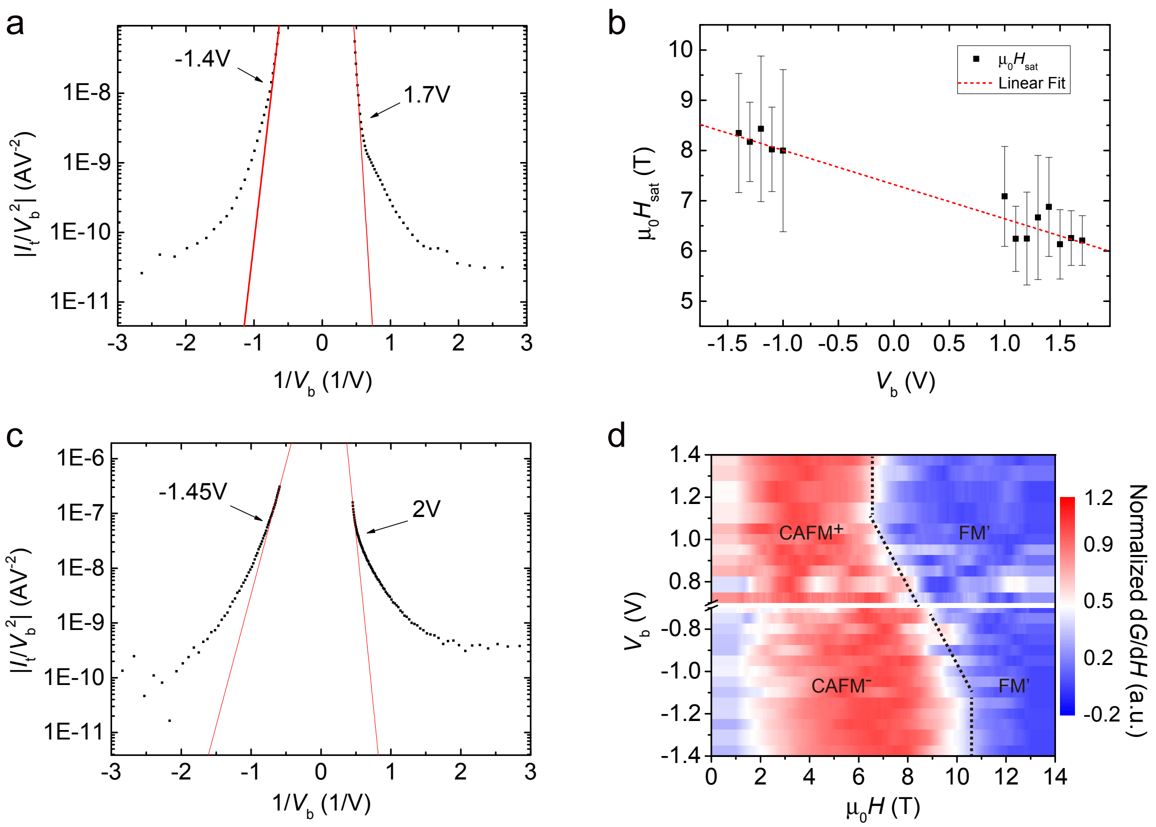

Supplementary Fig. 6. **FNT fitting and H_{sat} vs V_{b} characteristics of CCPS MTJs. a** and **c**, FNT fitting of CCPS MTJs 8L-S20 and 10L-S39, respectively. The fitting process allows us to differentiate the direct tunnelling regime from the FNT regime, which reflects the asymmetric work functions of the top and bottom FLG electrodes. **b**, H_{sat} vs V_{b} characteristics of CCPS MTJ 8L-S20. In between the binary saturating values for opposite V_{b} polarities, there is a quasi-linear growth of H_{sat} as a function of V_{b}. The error bars are defined as the $\pm 25\%$ variation of half-maximum points in dG/dH curve. **d**, Normalized dG/dH 2D contour plot for 10L-S39, in which the dichotomic H_{sat} plateaus for $V_{\mathrm{b}} > 1.1$ V and $V_{\mathrm{b}} < -1.1$ V, and the quasi-linear H_{sat} vs V_{b} relation in the transition areas are distinctive to see. Here, H_{sat} is defined by the border line between the red and blue zones, the latter corresponds to the **E**-enforced FM' state.

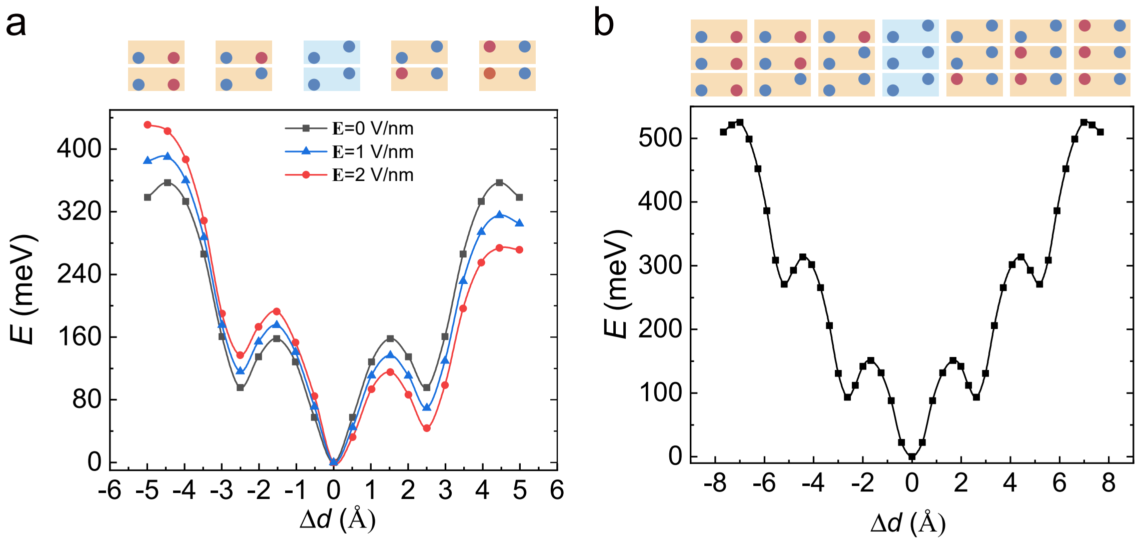

Supplementary Fig. 7. **Energy potential diagram of bilayer and trilayer CCPS. a**, Energy potential diagram of bilayer CCPS under different external electric field setpoints. Here we include the stripe-AFE ground state, the lowest metastable state 2L-mAFE-I, and the FE state. It is clear that the latter two states are not accessible even for an unphysical electric field of 2 V/nm. **b**, Energy potential diagram of trilayer CCPS, showing the stripe-AFE ground state, the lowest metastable state 3L-mAFE-I, the combination of 3L-mAFE-I and 3L-mAFE-II, and the FE state.

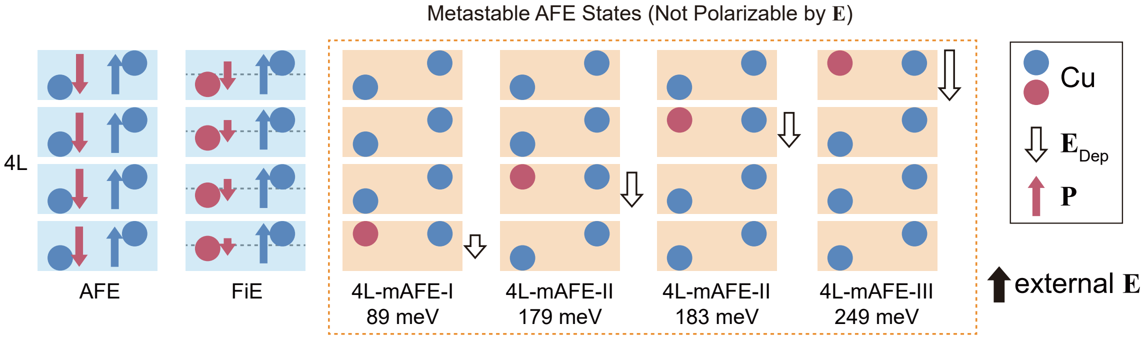

Supplementary Fig. 8. **Lattice model representation of the different energy states for quadruple-layer CCPS.** From left to right, we show the stripe-AFE ground state, the **E**-enforced FiE stae, and four energy inaccessible metastable AFE states, namely 4L-mAFE-I, 4L-mAFE-II, and 4L-mAFE-III for flipping the anti-parallel AFE stripe domain within different MLs.

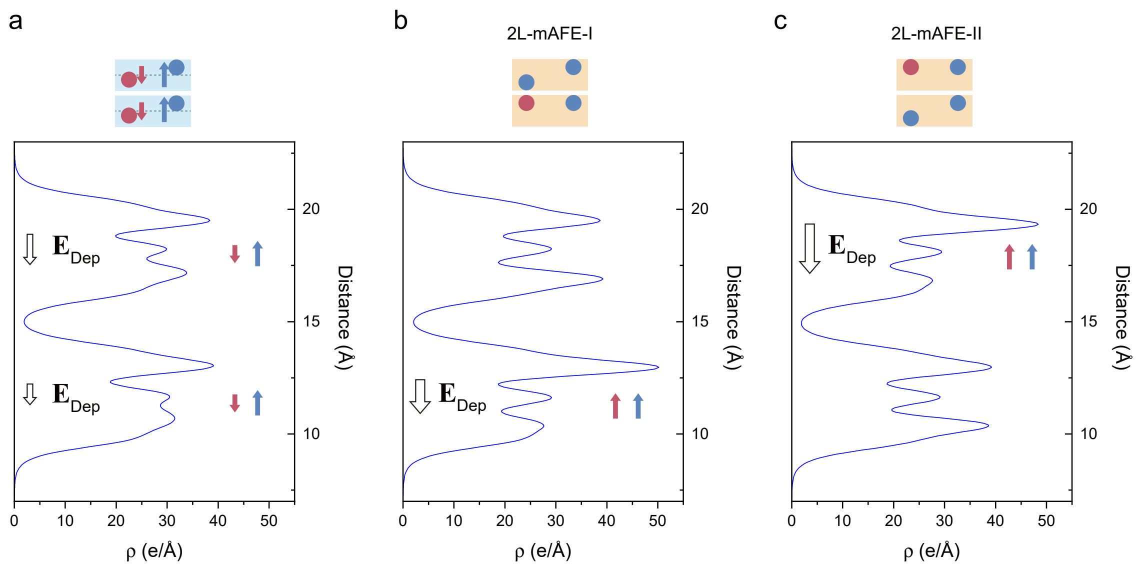

Supplementary Fig. 9. **Layer-dependent depolarization effects in bilayer CCPS. a** DFT calculated layer-dependent charge density distribution of bilayer CCPS in response to the same upwards AFE stripe domain displacements of 0.5 Å. The calculation results clearly demonstrate that the electron density in the bottom ML is more asymmetrically distributed towards the vdW interface when compared with the top ML, suggesting that the former is subjected to a substantially lower depolarization field due to extra dipole screening provided by the vdW interface. Similar trend is also confirmed for the metastable AFE states, as shown in (**b**) for 2L-mAFE-I and (**c**) for 2L-mAFE-II. For the lowest energy metastable AFE state 2L-mAFE-I, the flipped anti-parallel stripe domain has much lower energy and thus, a higher electron density, due to effective screening of the neighbouring AFE ML. Note that for 2L-mAFE-I and 2L-mAFE-II, the energy barrier maximum located at the Cr atomic plane is too steep to be crossed by Cu^+ ions.

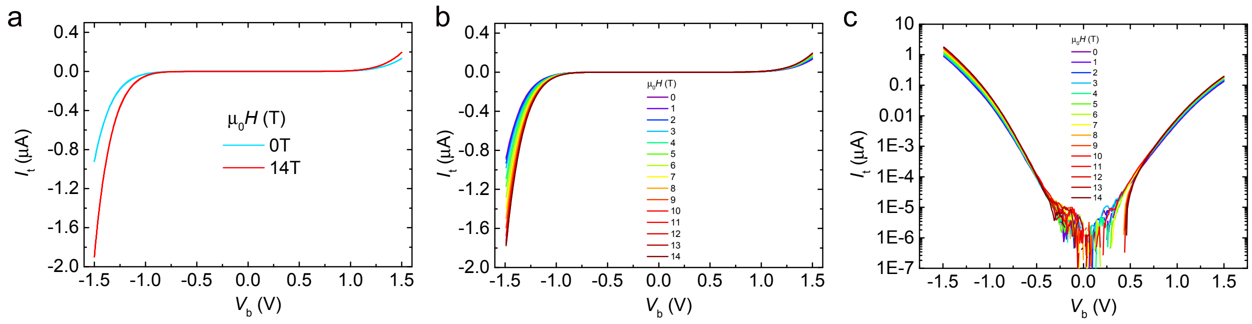

Supplementary Fig. 10. ***H*-dependent $I_t - V_b$ measurements of CCPS MTJs.** **a**, Two complete $I_\mathrm{t} - V_\mathrm{b}$ scanning loops of the CCPS MTJ 10L-S39 for $H = 0$ and 14 T respectively. Note that there is no hysteresis behaviour when V_b is ramped by $0 \rightarrow V_\text{b-max} \rightarrow -V_\text{b-max} \rightarrow 0$. **b**, H-dependent evolution of $I_\mathrm{t} - V_\mathrm{b}$ characteristics of 10L-S39 when H is slow-scanned from 0 T to 14 T with a step of 1 T. **c**, Same as (**b**), but in a logarithmic scale. The log scale plot proves the absence of FE or metastable-AFE transition related hysteresis behaviour.

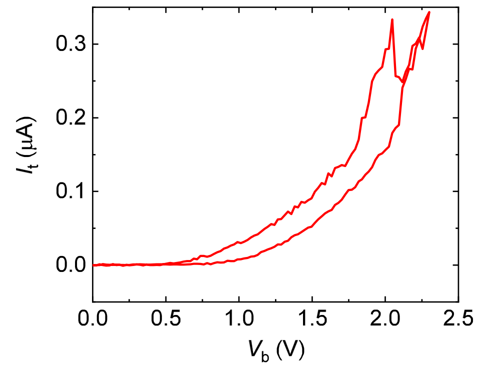

Supplementary Fig. 11. **Breakdown test of an octuple-layer CCPS MTJ 8L-S38.** For all measured CCPS MTJs, the upper limit of voltage breakdown limit is below 1.5 V/nm. Note that for 8L-S38, there is a build-in potential of 0.5 V. In general, the breakdown voltages are larger for thicker CCPS channels, and for smaller tunnelling cross-section areas. Nevertheless, 1V/nm is accessible for MTJs with ultra-narrow graphene electrodes, as shown in Figure 1e of the main text.

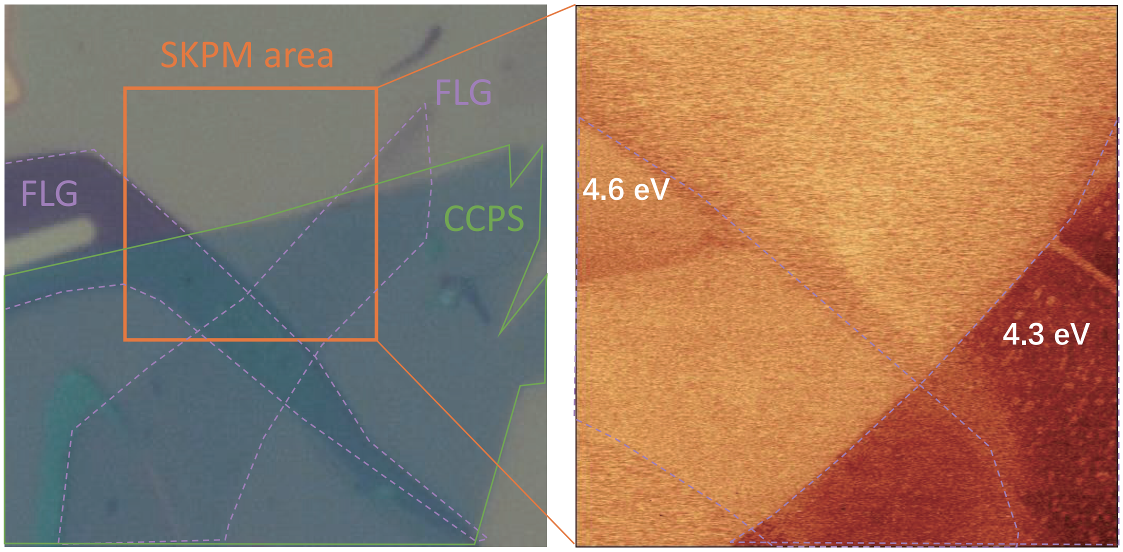

Supplementary Fig. 12. **SKPM measurements of a 7L CCPS tunnelling device.** The FLG electrodes and the CCPS channel are outlined by purple dashed line and green solid line, respectively, while the SKPM area is indicated by the orange square.

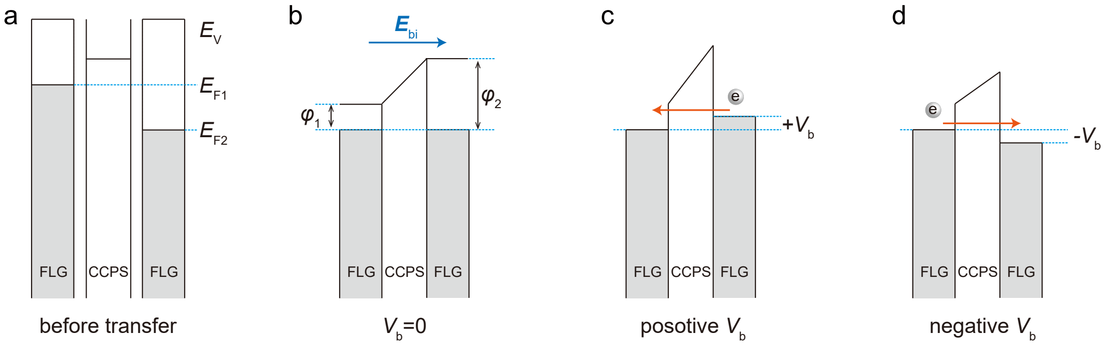

Supplementary Fig. 13. **Schematic illustration of build-in potential effects on the tunnelling current.** **a**, top and bottom FLG electrodes with different work functions before contacts. **b**, Build-in potential ($\mathbf{E}_{\rm bi}$) produced by the work function difference after the MTJ fabrications. **c** and **d**, Tunnelling electrons experience asymmetric barrier when applying opposite-polarity $V_{\rm b}$ with the same magnitude. Due to the existence of a build-in potential, the absolute values of tunnelling current should not be used as a criterion to differentiate different magnetic phases. However, the field-saturation point $H_{\rm sat}$ is only dependent on the interlayer AFM coupling energy $J_{\perp}$ and the layer numbers of thin-layer CCPS, which allows the FiE-interlocked MEC parameter to be precisely measured.

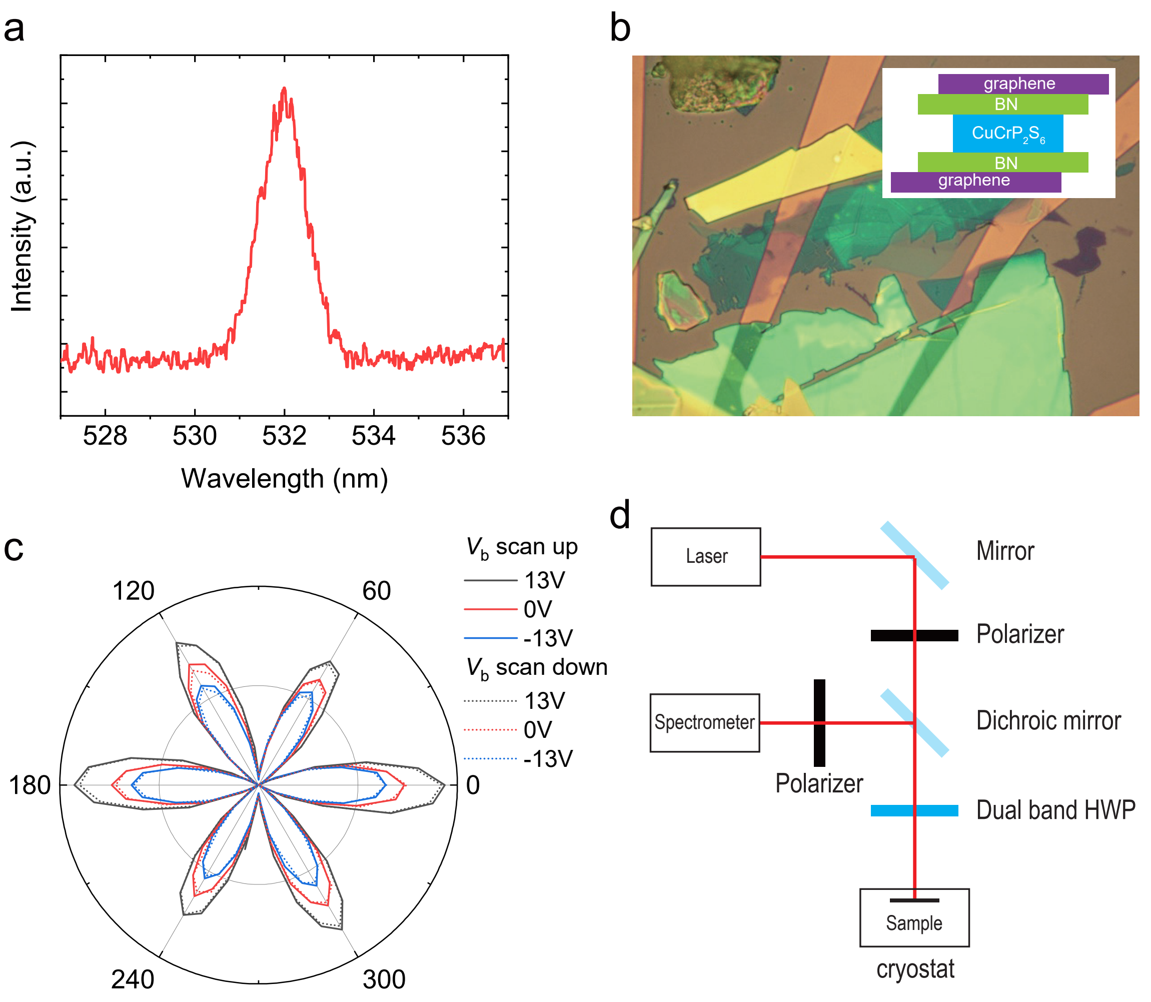

Supplementary Fig. 14. SHG measurements of thin-layer CCPS. a, SHG spectrum of an octuple-layer CCPS flake upon excitation of 1064 nm laser. **b**, An septuple-layer CCPS device with double BN encapsulations and top and bottom graphene electrodes for applying electric field. **c**, Polarization-dependent SHG of few-layer CCPS at 300 K under different V_b. Note the six-fold symmetry of SHG induced by paraelectric Cu^+ ion rearrangements within the CuS_6 vdW cages. **d**, Schematic of the optical SHG experiment setup.

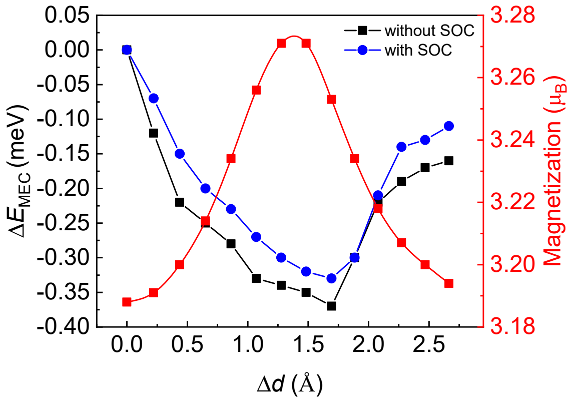

Supplementary Fig. 15. Monotonic dependence of ΔE_{MEC} on Δd, with Cu^+ ion displacements towards the energy saddle point (the Cr atomic plane). It is clear that spin-orbit coupling does not play a significant role in the FiE-interlocked MEC mechanism, which is not surprising due to the prevailing octahedron crystal field for Cr ions.

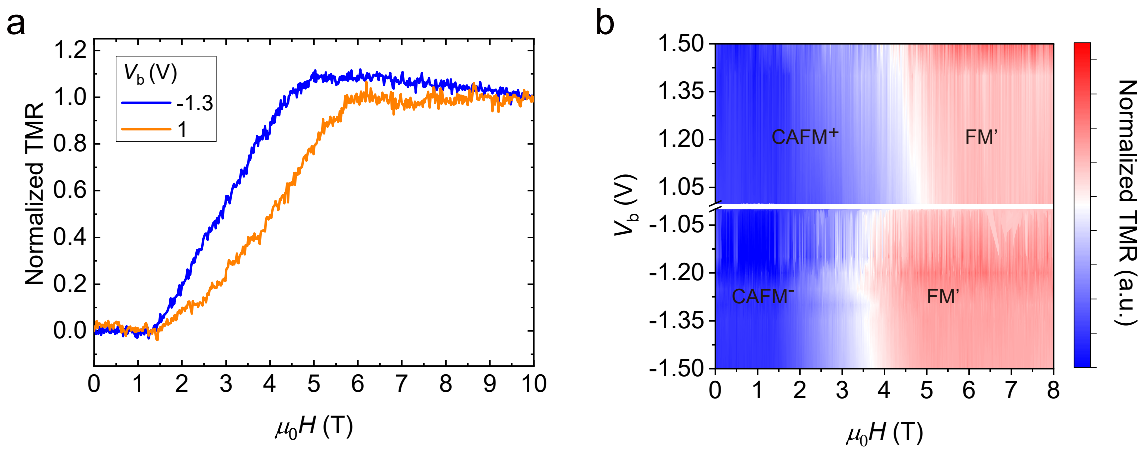

Supplementary Fig. 16. Ultra-wide V_{b}-tunability of the FiE-interlocked MEC in a quintuple-layer CCPS 5L-S32. a, Normalized TMR vs $H_{\|}$ curves of MTJ 5L-S32 under opposite-polarity V_{b} setpoints. **b**, 2D contour plot of the normalized TMR vs V_{b} and $H_{\|}$ of 5L-S32, showing dichotomic H_{sat} values for opposite-polarity V_{b}.

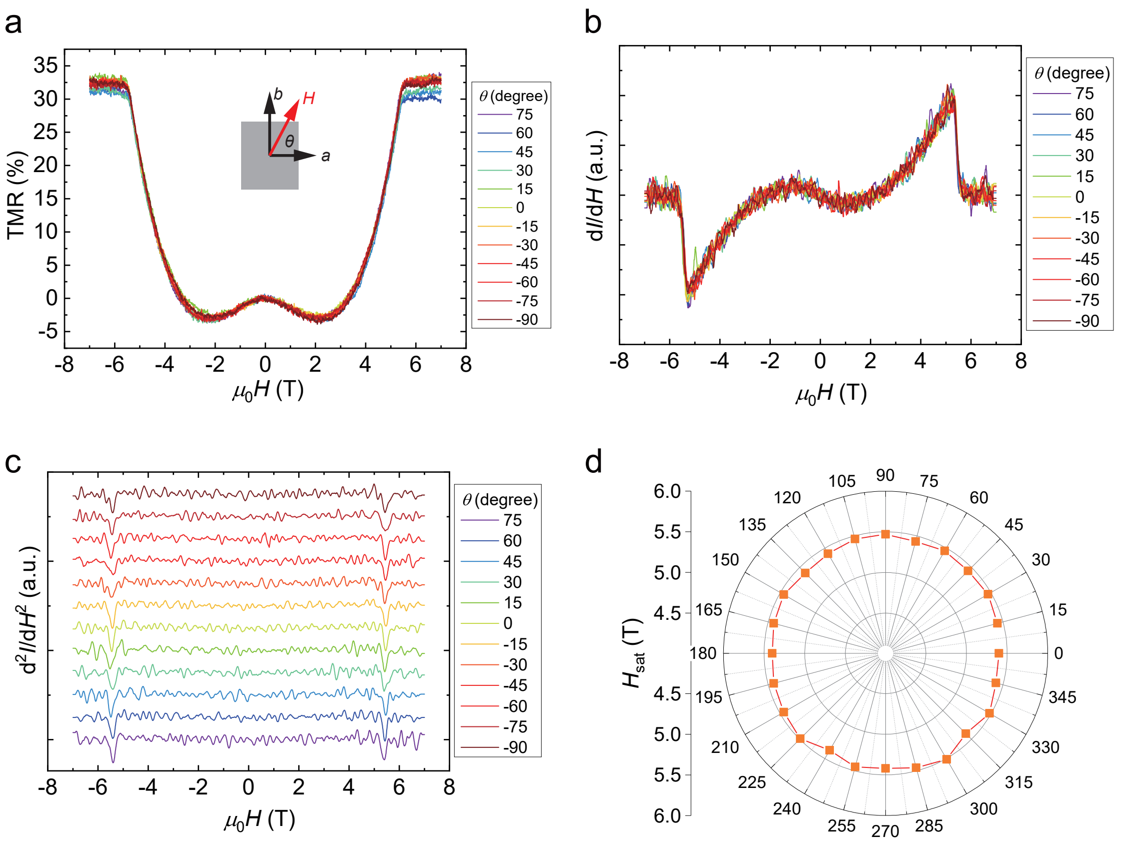

Supplementary Fig. 17. **TMR measurements of CCPS 6L-S37 under in-plane H rotation.** **a**, Measured TMR versus H curves with different in-plane angle setpoints. **b**, $\mathrm{d}I/\mathrm{d}H$ analyses of the TMR spectra, revealing identical behaviour for different angle setpoints. **c**, $\mathrm{d}^2I/\mathrm{d}H^2$ analyses of the TMR spectra, confirming the $\mathrm{d}I/\mathrm{d}H$ analyses. **d**, Polar plot of H_{sat} versus in-plane H angle, proving the easy-plane magnetocrystalline anisotropy of thin-layer CCPS.

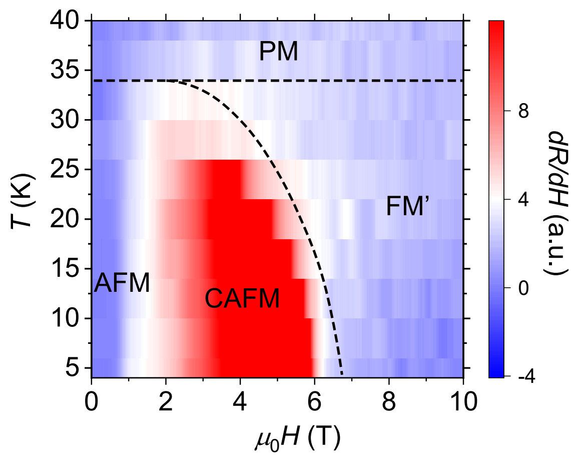

Supplementary Fig. 18. **2D contour plotting of the first derivative of TMR (dR/dH) vs $H_{\parallel}$ and T for 7L-S21.** By taking the derivative, the magnetic phase diagram of odd-layer CCPS is clearly revealed, which is essentially the same of even-layer CCPS except the field-induced perfect collinear zone below 2 T.

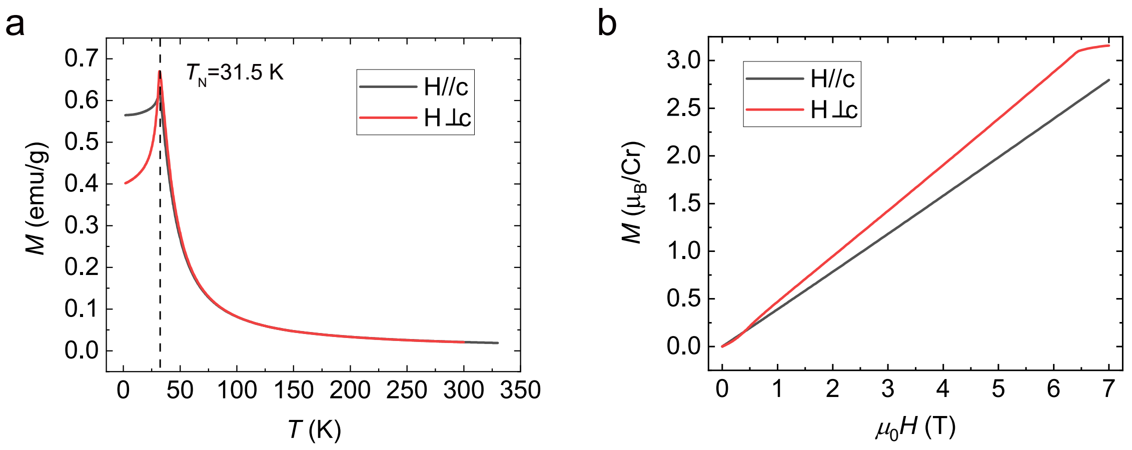

Supplementary Fig. 19. **Magnetic properties of bulk CCPS. a**, T-dependent magnetization characteristics of a CCPS single crystal measured with a small H=1000 Oe. Note that the Néel temperature T_{N} = 31.5 K is retained for thin-layer CCPS down to the bilayer thickness. **b**, Magnetic moment versus in-plane and out-of-plane H measurements of CCPS single crystals. The saturation value of $\sim 3\mu_B$/Cr for $H \perp c$ proves the easy-plane magnetocrystalline anisotropy of CCPS.

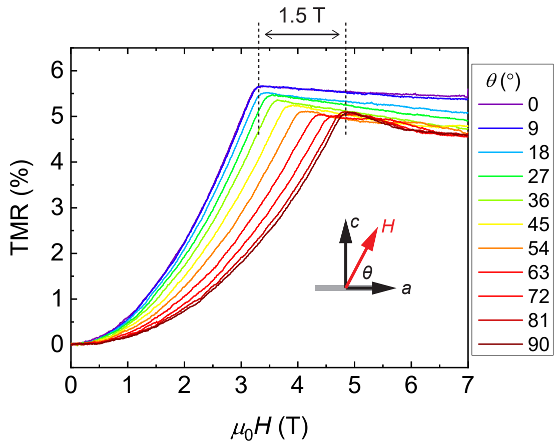

Supplementary Fig. 20. **TMR measurements of CCPS BL-S40 with in-plane to out-of-plane H rotations.**
